# Trust, medical expertise and humaneness: A qualitative study on people with cancer’ satisfaction with medical care

**DOI:** 10.1111/hex.13171

**Published:** 2021-02-02

**Authors:** Susanne Blödt, Jacqueline Müller‐Nordhorn, Georg Seifert, Christine Holmberg

**Affiliations:** ^1^ Institute of Social Medicine and Epidemiology Brandenburg Medical School Theodor Fontane Brandenburg an der Havel Germany; ^2^ Institute of Public Health of the Charité – Universitätsmedizin Berlin Humboldt‐Universität zu Berlin Berlin Institute of Health Berlin Germany; ^3^ Department of Pediatrics Division of Oncology and Hematology, Charité – Universitätsmedizin Berlin Humboldt‐Universität zu Berlin Berlin Institute of Health Berlin Germany; ^4^ Faculdade de Medicina Departamento de Pediatria Instituto de Tratamento do Câncer Infantil (ITACI) Universidade de São Paulo São Paulo Brazil; ^5^ Faculty of Health Sciences Brandenburg Brandenburg Medical School Theodor Fontane Potsdam Germany

**Keywords:** breast cancer, good care, patient narratives, prostate cancer, qualitative research

## Abstract

**Background:**

Understanding peoples’ evaluations of their health care is important to ensure appropriate health‐care services.

**Objectives:**

To understand what factors influence peoples’ satisfaction with care and how interpersonal trust is established between doctors and cancer patients in Germany.

**Design:**

A narrative interview study that included women with a diagnosis of breast cancer and men with a diagnosis of prostate cancer. A question‐focused analysis was conducted.

**Setting and participants:**

Interviewees were sought across Germany through self‐help organizations, clinics, rehabilitation facilities, physicians and other health‐care professionals, in order to develop modules on experiencing cancer for the website krankheitserfahrungen.de (illness experiences.de).

**Results:**

Satisfaction was related to the perception of having a knowledgeable and trusted physician. Trust was developed through particular interactions in which ‘medical expertise’ and ‘humaneness’ were enacted by physicians. Humaneness represents the ability of physicians to personalize medical expertise and thereby to convey working in the individual's best interest and to treat the patient as an individual and unique human being. This was fostered through contextual and relational factors including among others setting, time, information transfer, respect, availability, profoundness, sensitivity and understanding.

**Conclusion:**

It was the ability to make oneself known to and know the patient in particular ways that allowed for satisfying care experiences by establishing interpersonal trust. This suggests the importance of conceptualizing the doctor‐patient relationship as a fundamentally reciprocal human interaction of caregiving and care‐receiving. At the core of the satisfying care experiences lies a doctor‐patient relationship with a profoundly humane quality.

## 1 INTRODUCTION

Trust is a fundamental aspect of human interactions in contemporary societies. As Giddens^1^ has suggested, trust is a precondition of modern society, where so much is outside of an individual's realm of knowledge and expertise, and where dependence on technical systems is large. To maintain trust in such systems, regulatory structures are employed.^2^ For example, for effective health‐care delivery trust in the medical system is a precondition for individuals to seek help there. As a first step to use the health‐care system, there has to be general trust in the system, something that Luhman has called institutional trust.^3^ Measurable quality and performance indicators, certified treatment centres, publicly available hospital reports and licensing for physicians are all there to ensure and maintain institutional trust.^2^ But in addition to this, interpersonal relationships with health‐care workers and the individual experience of care can lead to what Luhman has called interpersonal trust, which can in turn strengthen or weaken institutional trust.^3^

Institutional and interpersonal trust are interlinked in webs of interaction and are difficult to separate from one another,^4^ particularly in a system as complex as health care.

Patients entrust themselves in the care of institutions and physicians. Patients per definition are in a vulnerable position and are dependent on the knowledge and experience of health‐care workers, particularly in the case of life‐threatening situations. In the literature, several concepts of trust in health care exist.^2^, ^5^, ^6^, ^7^, ^8^ They share a common understanding that a vulnerable situation, such as receiving a cancer diagnosis, is a moment where trust is necessary, and entails the assumption that the physician is acting in the patient's best interest.^9^ Within the system, the physician is the role which is endowed with the power to help the patient, thus the doctor‐patient relationship, while crucially important,^10^ is by definition characterized by an imbalance, in which interpersonal trust comes into play. Studies that have investigated the importance and effect of trust in a physician have found that a trusting relationship can improve patients’ quality of life^11^ and appears to be fundamental when characterising good patient‐doctor interactions.^9^, ^10^, ^12^, ^13^, ^14^ A positive and supportive relationship with a physician is associated with a decrease in patients’ emotional distress, better treatment adherence,^15^ better physical outcomes^16^ and higher patient satisfaction.^17^, ^18^ Thus, it is not surprising that trust in a physician and the provided health care seems to influence patients’ information needs and information seeking—as we found in a previous analysis of the role and meaning of health information for individuals’ experiences with breast, colorectal or prostate cancer.^19^ Our findings showed that information was important for individuals in the emotional management of their illness experiences. There also seemed to exist a link between information needs, satisfaction with care and relationships with the treating physicians in the interview data. It is this residual finding, which we aim to explore further in this article.

While there has been increasing focus in recent years on trust as the foundation of good patient‐physician interactions, little is known about how a trusting relationship comes about in patients’ experiences. In a review of existing qualitative studies on patient‐physician relationships, Ridd and colleagues found that a relationship is established by on‐going, longitudinal care from the same doctor and through particular consultation experiences.^10^ Yet as Hillen et al^20^ have pointed out, a key characteristic of contemporary health care is increasing specialization, which involves interactions with more and changing physicians, as one goes from one specialist to another. This is particularly true in cases of severe illness, such as cancer care. The authors contend that in the case of cancer care, in order for patients to trust their specialist physicians, fidelity (understood as a physician acting in the patient's best interest), caring, medical competence and honesty are key characteristics that the physicians need to display. But how does a patient evaluate her health care? What factors influence a patient's satisfaction with care? Related to this are the questions of how does a patient decide that a physician is acting in her best interest? Finally, how does a patient come to trust her physician?

In the following, we address these questions by investigating how people living in Germany who have had a diagnosis of cancer talk about how they came to trust their physicians and how this relates in the interviews to being satisfied with their cancer care.

## 2 METHODS

### 2.1 Design

A narrative interview study was conducted with women with a diagnosis of breast cancer and men with a diagnosis of prostate cancer.^19^ The interviews were collected between 2012 and 2013 for the purpose of developing modules for the health information website krankheitserfahrungen.de focusing on people's experiences of various diseases. Ethics committee approval was obtained from the University of Freiburg (EA/247/12) and was reported to the Charité – Universitätsmedizin Berlin ethics committee (EA4/053/12). Data collection and presentation on the website were funded by the German Federal Ministry of Health (NKP‐332‐041). The data analysis presented in this article was funded by the foundation Krebsallianz. Interviewees included in the presented analysis gave informed consent for the use of their interviews for research.

### 2.2 Data collection

This interview study used maximum variation sampling (with regard to age at interview, age at diagnosis, treatment, course of disease, socio‐demographic factors) to include as many different aspects of experiences with breast or prostate cancer as possible.^21^, ^22^ Interview participants were sought from different parts of Germany with the help of the research team, self‐help groups, health professionals, primary care clinics and rehabilitation centres. Interviews were either audio‐ or video‐recorded based on the preference of the interview partners. The latter also decided where the interviews were conducted. Most interviews took place at the homes of the interviewees. Interviewers had long‐term experience in qualitative data collection methods. The interviewer and interviewee were of the same gender (male‐male, female‐female). All interviews begun with the same question, with the aim of starting a narration: ‘Can you tell me how your life was when you first became aware of the signs/symptoms of cancer and how it went from there? Please take your time and tell me how one thing led to another’. After this initial narrative sequence, follow‐up questions were asked to elicit all relevant aspects of the illness experience. Follow‐up questions were based both on the initial information provided by the interviewee and on a literature review. Themes included were the diagnosis process, treatments, information seeking, family and partnership, communication, help and support, and living with a cancer diagnosis. The interviews ended with an open question asking what message the interviewee would want to give to fellow patients as well as to physicians.

### 2.3 Data analysis

Based on analysis of the interview data presented on krankheitserfahrungen.de, which was conducted for the previous article on the meaning of information in the illness experiences,^19^ we found that information needs, satisfaction with care and trust in the physician were seemingly interconnected. To investigate these first impressions in more detail, we conducted a secondary data analysis using a question‐focused approach for all of the interview material. The questions posed in relation to the material were: When and how do interviewees talk about satisfaction with their care? What are the circumstances interviewees describe when they talk about satisfaction with or trust in their physicians? Which attributes do they use to characterize their health‐care providers?

We were interested in patients’ emic views, as conveyed in the interview material, regarding what constitutes trust and satisfaction with care. We therefore built the data analysis iteratively in the following way. To get an overview of the material in relation to our questions, one of the research team members (SB) identified text passages through lexical searches, in which patients mentioned the words ‘physicians’, ‘care’, ‘trust’, ‘satisfied’ and ‘unsatisfied’. Terms used in the lexical search of the interview material from the men diagnosed with prostate cancer further included: ‘physician’, ‘surgeon’, ‘urologist’, ‘family doctor’, ‘therapist’, ‘senior physician’, ‘relief’, ‘university hospital’, ‘satisfaction’, ‘good’, ‘professor’, ‘best/good hands’, ‘recommendation’, ‘experience’ and ‘atmosphere’. The first author (SB) read all of the highlighted passages, as well as the entire interviews in order to ensure that other passages dealing with care but that did not use the search terms were not missed.

All identified text passages were first coded according to the question what was talked about in the sequence about care. Codes such as setting, practice, physician and others derived from the material were used. Then, text passages with similar codes were compared to one another, and codes were developed to characterize the care experiences and factors influencing them. In the final round, the codes were separated out in good and bad evaluations of care experiences and factors associated with those experiences. This procedure was initially done with nine interviews. Codes related to care were then organized into more conceptual categories according to the attributes displayed relating to satisfaction/dissatisfaction or good/bad care experiences. Categories and associated text passages relating to satisfaction/dissatisfaction were discussed (CH and SB) and grouped into elements and factors of good care. One core category emerged, around which other categories that had been developed from the initial codes could be grouped, thus creating first the factors, than the associated elements associated with care experiences. As analysis pursued, factors and elements were refined and further condensed. This process was complete following the analysis of eight additional interviews. The remaining 25 interviews were read in detail, but this did not further change the core category.

The same process with the same words was carried out for the interviews with women diagnosed with breast cancer. Due to the different cancer entity, however, the terms ‘gynaecologist’, ‘psycho‐oncologist’, ‘breast cancer centre’ and ‘alternative practitioner’ were added to the filter, while ‘urologist’, ‘surgeon’ and ‘senior physician’ were deleted. The selected text sequences were coded separately for five interviews. Then, the same iterative process of factor development was conducted as described above. Fourteen more interviews were coded until data saturation was achieved. The remaining 24 interviews added no additional elements or factors. The analysis for the two disease entities were discussed in team meetings between the last author (CH) and SB. As the results for the research questions were similar, they could be collapsed in more general elements and factors. Consolidated criteria for reporting qualitative research (COREQ) was used as a reporting guideline.

## 3 RESULTS

### 3.1 Sample description

The sample consisted of 43 women with a diagnosis of breast cancer and 42 men with a diagnosis of prostate cancer. The socio‐demographic characteristics of the interviewees are published elsewhere.^19^ Age and time since diagnosis varied among the participants. The age range at diagnosis was 25‐71 years in the female sample and 47‐74 in the male sample. Time since diagnosis varied from five months to 21/15 years for the women and men, respectively.

### 3.2 Satisfying care experiences from the patient's perspective

#### 3.2.1 Factors influencing patients’ satisfaction with care

**FIGURE 1:**
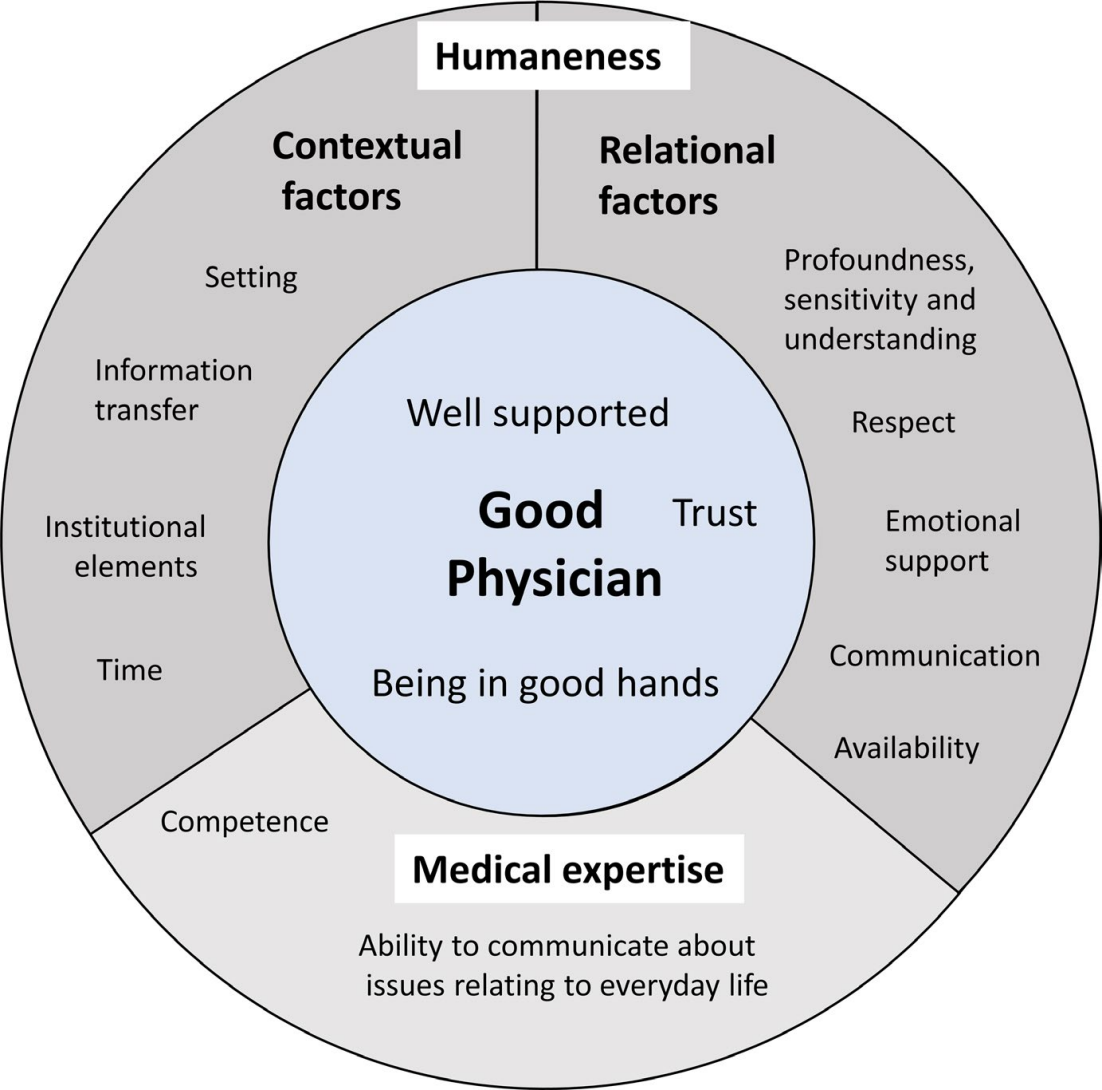
Elements and factors of good care

In the following, we will first address the question of what factors influenced interviewees’ evaluation of care (Figure 1). All text passages that were identified as referring to satisfying, positive or good care experiences spoke about a physician who was trusted and perceived as knowledgeable. This led to the core category of the ‘good physician’, which we placed at the centre of the elements that constitute good or satisfying care. Interviewees who were satisfied used terms such as ‘being in good hands’ and ‘being well cared for’ to describe their relationship with their treating physician. In the identified text passages, the physicians were characterized as being experts in their field and had the ability to sense patients’ concerns and, without glossing over the situation, could give them emotional support in situations in which they felt desperate.

For some interviewees, the knowledgeable physician was sufficient in their evaluation of their care. Perceiving the physician as knowledgeable presented the foundation of being well cared for. The second key factor displayed in the text passages was what we call ‘humanness’. It stands for the ability of the physician and the health‐care institution to recognize and treat the patient as an individual and unique human being. While this key factor is most importantly an attitude towards the patient, it can be fostered by a range of factors both contextual and relational. C*ontextual factors* included setting, information transfer, institutional factors and time. R*elational factors were composed of* sensitivity and understanding (in examinations and information provision), respect, communication, emotional support and availability.

#### 3.2.2 Medical expertise

Interviewees cited different attributes that characterize a knowledgeable physician. They talked about the competency and reputation of a physician, their experience (number of years practiced or number of procedures performed), holding the title of professor, having many contacts, being affiliated with a university centre or certified cancer centre, being well‐known and respected by other physicians, or giving a good performance at an information event.

It was the head physician of the local clinic who was giving a presentation. A very likeable man, and I asked him how many surgeries he had conducted and it sounded quite reasonable. At the time 40, 50 surgeries a year was a good basis to know: this man has experience. (Male, aged 72, 11 years after prostate cancer diagnosis)

I would only go to a certified breast cancer centre, and not to any old clinic that the physician transfers me to because I cannot be admitted elsewhere. And 2‐3 weeks doesn’t play a role, it’s not contagious. (Female, aged 63, 16 years after breast cancer diagnosis, recurrence 12 years ago)

#### 3.2.3 Humaneness

Humaneness was composed of both contextual and relational factors.

#### 3.2.4 Contextual factors

The setting in which conversations and interactions took place was mentioned as an aspect of good care. A pleasantly arranged space was generally appreciated by the interviewees; for instance, a quiet space and a chair to sit on were linked to a welcoming setting, especially when a diagnosis or laboratory results needed to be addressed. Interviewees complained about physicians who delivered bad news in the hallway or simply in passing on their way somewhere else.

Because this physician did not break the news in the physician’s room, but in the hallway, just like a concierge who is passing by and says “sweep up that corner”. (Male, aged 64, 3 years after prostate cancer diagnosis)

Similarly, the importance of information transfer was emphasized. Whereas most interviewees said that they wanted to receive facts and hear the ‘truth’ about their diagnosis, and disliked it when physicians beat around the bush, others perceived the way in which the diagnosis had been disclosed to them as cruel, and complained that the physician had been insensitive.

[I do not want] to sit yet again with the physician, who again is telling me that everything is normal and to wait a bit longer, it will get better. … I would like once in a while to hear the truth. Namely the truth that it won’t get better. That it is like it is… I want to know the truth, for me, because then I can say: ‘This is how it is and now I can look for a way to deal with it’. (Female, aged 51, 1 year after breast cancer diagnosis)

‘Yes, this looks terrible, completely bad’. ‘What’s bad?’ I said. … And then he gave me the sealed letter and I thought, I left and then I thought, I want to know it now. (Male, aged 64, 3 years after prostate cancer diagnosis)

But in the hospital, they need to say: ‘We will do this and that, and this and that is necessary’. Like with the bone scintigram, I did not know that I had received a radioactive injection and that I would need to wait four hours for the diagnosis, and before that I was placed in such a position so that I could see the x‐ray. I saw a lot of black marks on my lungs. I thought: ‘Eh, it’s already progressed so far, my whole lungs are full’. And that’s not right, that they leave people alone with their own worst fears. (Female, aged 44, 3 years after breast cancer diagnosis)

The institution can also facilitate or hinder a humane atmosphere, in terms of its organization of care delivery and the care environment. In general, interviewees positively acknowledged good organizational structures, such as when scheduled appointments took place on time, when the next steps of the treatment or diagnostic process were made clear, and when disease management was planned ahead, which allowed patients to arrange their own plans accordingly. Furthermore, the continuity of doctors and contact persons was an important element in building trust.

The physician was on holiday and his secretary scheduled my appointment for his first day back. I was there, we talked, we liked each other, and he said: ‘In 14 days you have an appointment if you like’. I appreciated that everything ran smoothly. (Male, aged 69, 1 year after prostate cancer diagnosis)

And very, very lovely people, always the same ones, who gave the treatment, with a warm welcome, always in a good mood, friendly without exaggerating, very friendly people. If you had any requirements you could talk to the physician, appointments were always on time. It really was very lovely. (Female, aged 59, 1 year after breast cancer diagnosis)

He just said: ‘I’ll call them and make an appointment for you’. This was easier for me this way. And I could allow myself to let go and to say: ‘Go ahead’. And I just went to the appointments that I had. This was important for me, that there was someone pulling the strings. (Female, aged 53, 1 year after breast cancer diagnosis)

The experience of time was a crucial factor for the entire care experience. This included, for example, the time given for conversations with the doctor and for asking questions. Time was also crucial for some interviewees when it came to making a decision and/or ‘getting ready’ for a treatment.

The urologist said that it was only a few tumour cells and that I could take my time with the surgery. He addressed my needs very well. (Male, aged 61, 3 years after prostate cancer diagnosis)

I always had the choice, and I found this very important, also from the doctor, that I could decide. I didn’t have to do everything so quickly. Also before the first operation, I asked: ‘Do we need to go fast with this?’ And the doctor said: ‘No, your cancer is not aggressive, take your time. Accustom yourself to the thought. Think about it for a while and then you can tell me a date’. (Female, aged 60, 2 years after breast cancer diagnosis)

This physician who set the wire into the tumour, he explained much more to me. He really took his time, even though he was short on time, because there were people in the waiting room. He said: ‘No, you are sitting here. You have questions now and every question will get an answer’. (Female, aged 60, 20 years after breast cancer diagnosis)

#### 3.2.5 Relational factors

The profoundness, sensitivity and understanding exhibited by a physician when performing a consultation guided interviewees’ perceptions of a humane care experience. Almost all of the interviewees mentioned at one point during the interview how important it was that the physician supported the trajectory that they themselves chose and respected their decision, also in cases where their decision differed from the physician's recommendation. Emotional support was important for many interviewees, which involved having their concerns taken seriously and being adequately addressed. Availability, namely the possibility to reach the particular trusted physician when necessary, also guided patients’ perceptions of humaneness in care. Thus, communication as the way in which physicians interacted with patients, was crucial. Being a ‘good physician’ was linked with a physician who listened to patients’ needs, who were open‐minded to their questions, and patient enough to answer them.

What was good was that my urologist, who advised me completely based on the guidelines of the urological society or whatever it’s called, when I made my decision and said ‘No surgery for me’, he said ‘Good, it’s your decision, I support you. We will do blood tests every three months, we’ll take it easy’. (Male, aged 48, 1 year after prostate cancer diagnosis)

And that they [physicians] give you the feeling that they are dealing with a human, not with a number. I also experienced the feeling that as a human being you are taken seriously and recognised. (Female, aged 66, 18 years after breast cancer diagnosis)

He had plenty of patients in the waiting room, it should have been quick, and still he gave me his attention. … He has interest, is fully present and is aware of who is sitting there. This is what I need, that I am recognised (laughs) and not only: ‘There sits a prostate on the stage, of course we’ll get it out’. (Male, aged 48, 1 years after prostate cancer diagnosis)

#### 3.2.6 Coming to trust a physician

The text passages in which interviewees talked about feeling in good hands or trusting their physician often contained descriptions of particular encounters that explained why the patients had a good feeling about entrusting their well‐being and treatment to a particular physician.

The presented quotes represent typical text passages in which a patient's satisfaction with care was connected to a particular physician.

One thing I have learned, and that is what this man [physician at the university hospital] said. He said: ‘The methods are all very similar with risks that are comparable. The dexterity of the surgeon is comparable, only the experience is different. And I have done this surgery over 2000 times’. This means that with this physician you are in good hands, he knows what he’s doing. (Male, aged 69, 1 year after prostate cancer diagnosis)

From the first second I had a feeling of wellbeing, because she [the senior physician] was listening to me, she was facing me. She examined me herself and said: ‘*I am palpating this, I want to make my own picture*’. I would have wished this for the first genetic test (…). The starting point came from the senior physician, who sent us [the interviewee and a woman with breast cancer diagnosis in the same age] in the together for this first examination, where the lymph nodes were examined. *And she also gave us a double room in the hospital*. She organised that. I simply have to say that I have great respect for that. She arranged that in a very human way and from then on we both really went through the whole disease together, which is extremely helpful. (Female, aged 54, 1 year after breast cancer diagnosis)

What helped me a lot was that this female physician [at the breast cancer centre, second opinion] was very empathetic and counselled me well. And she said at the very beginning – and this eased the whole thing, that she said – ‘You have breast cancer, this is the first thing. And the second is that it is curable, it is a hard path’. (Female, aged 52, 1 year after breast cancer diagnosis)

It was also typical that the particular physician stood out from other physicians; they were able to resonate with the patient in such a way that the patient felt themselves to be ‘in good hands’. In the recollection of the prostate cancer patient above, the key point for him was the surgeon's statement regarding the number of times he had performed the procedure. Such an empirical measurement of experience on the institutional level is currently used in certification programs and hospital assessments as quality indicator. The main German prostate cancer self‐help organization was politically active in the attempt to enact such quality indicators for institutions in prostate cancer care at the time of the interview. It may have been for this reason that this particular statement by the physician helped the patient to feel that he was in ‘good hands’ and made the physician stand out. We would argue that the physician in this case personalized a standard quality indicator from the institution to himself so that the patient, for whom this was important, could put his trust onto the particular physician. Interpersonal trust was thus established, a necessity for a good care experience.

Other factors that enabled a physician to stand out and earn trust were thoroughness and a communication style that made the interviewees feel recognized and seen. This also gave the physician a personal touch that distinguished him or her from other physicians. This is conveyed in the second text passage above from the interviewee with breast cancer, when she describes how the physician performed an examination again in her practice in order ‘to make her own picture’. Here, the description of the physician taking the time to see the patient as an individual, and to form her own opinion about the diagnosis and tests, enabled the patient to trust her. In the final example above, the interviewee spoke about how she perceived the physician to be honest, not only in terms of giving the facts, but also in admitting that the path will be a difficult and challenging one, while combining this with reassurance and hope.

The above text passages describe physicians who were able to present themselves in such a way that the patients recognized them as special and felt that they would act in their best interest. This, we argue, is crucial in terms of how patients come to trust their treating physician, which in the interviews was the most important aspect for experiencing satisfying care.

## 4 DISCUSSION

When pursuing medical treatment, patients must entrust themselves to the care of an abstract system.^1^ They also need to trust the representatives of this system. According to our findings, in cancer care the physician seems to be the central character (or representative of the system), and the physician‐patient relationship appears to be most important in terms of enabling a satisfying care experience. It was the ability to relate to the patient in a personal manner that stands at the core of being a good physician. Organizational factors could support the experience of satisfying care.

For the interviewees, it was critical that they had the feeling that their physician had expertise and was knowledgeable; this has been highlighted as an important aspect of care in a range of studies.^20^, ^23^, ^24^, ^25^ Based on their methodologies, however, these other studies could not investigate how patients come to the conclusion that a physician is knowledgeable. Patients can seldom judge from a professional standpoint whether a physician is good at what they do, thus they need proxies upon which to base such judgements. In our study, such proxies for being a good physician, understood as being a *knowledgeable* physician, were largely related to the quality indicators used for the certification of health institutions, including measurable quality and performance indicators, certified treatment centres, publicly available hospital reports and licensing for physicians.^2^ Here, the interrelatedness of institutional and interpersonal trust once again becomes clear. In a recent review on patient satisfaction, health‐care service quality indicators also seemed to correspond to the influential determinants of patient satisfaction, with the health providers’ interpersonal skills being the most important.^26^

We argue that the way interviewees decided that a physician had the necessary expertise for their treatment depended on the physician's ability to make herself ‘known’ to the patient. The text passages show narratives of encounters in which a physician was able to stand out in a particular way. We call this ‘personalization’. It presented a firm base upon which to build a trusting relationship based on honesty, working in the patient's best interest and medical competence. In addition to these aspects of trust, Hillen et al (2012) identified ‘caring’ to be important in cancer patients.^9^ The authors used the term to label caring behaviours that physicians displayed in relation to the patients’ personal well‐being. This relates to what we have labelled ‘humanness’. We chose the broader term as it signifies the fundamental aspect of the human condition. Caregiving, Kleinman (2015)^27^ argues, is a ‘defining condition of what it means to be human’. Caring as an integral and crucial aspect in physicians’ work has been largely neglected in the theorising about care. Indeed, care and caregiving have an important place in social science and feminist research that has investigated the work provided by nurses, family members and particularly women. Caring as a fundamental human condition has become remarkably absent from the medical sciences and debates of health‐care delivery. When the interviewees for this study were satisfied with the care they had received, having the feeling of being cared for and recognized by their physician was crucial.

Caregiving is relational and reciprocal. Cornwell^28^ has summarized how relational aspects—such as listening to and spending time with a patient, using accessible language, treating the patient as an individual, not labelling the patient, giving the patient the feeling of being informed and involved in care and treatment options, and having access to knowledgeable professionals and continuity of care—as well as functional aspects—such as efficient processes, positive outcomes and receiving information about treatments and technologies—all matter to cancer patients.

The importance of relational elements for patients in terms of judging their health‐care provider has been shown elsewhere.^23^, ^24^, ^25^, ^29^ In this article, we show how relational elements are part of the assessment through which a physician becomes trustworthy to a patient. In fact, one could argue that if we understand caregiving and care‐receiving as part of the fundamental human condition as reciprocal, we may go further in our understanding of the doctor‐patient relationship. While patients are in a sense vulnerable and need to trust, they will in retrospect evaluate the care experience in respect to their being treated in a fundamentally humane way. At the core of the satisfying care experiences lies a doctor‐patient relationship with a profoundly humane quality. It was this quality that can be found in the text passages we identified. Contextual and relational factors support or are inherent in such a fundamental attitude of human to human interaction. The attributes interviewees used to characterize a good physician, such as his or her knowledge and medical expertise, time factors, sensitivity and understanding, respect, emotional support, communication and availability all allude to this. Bickel et al^23^ have written that the perception of care is a very individual assessment. However, we could identify some processes that were implicated in a patient's experiences, when they talked about satisfactory care for their cancer.

### 4.1 Limitations

Our paper has several limitations that need to be considered when interpreting the results. The interviews with women with a diagnosis of breast cancer and men with a diagnosis of prostate cancer were conducted using the same interview guide—with slight adaptations due to the cancer entity—but by two interviewers: a male interviewer for the men and a female interviewer for the women. Although both interviewers followed the same interview guide, differences in the interview style due to gender and personal characteristics between interviewers could have had an influence on the interviews and may explain in part why some issues only came up in one of the two interview groups. Another limitation is that at the end of the interviews, the interviewees were asked in an open question what message they would like to give to physicians, and this might in part explain why physicians were so crucial in the interviewees’ narrated care experiences, and why other health‐care providers were not mentioned regarding good care experiences. Nevertheless, the importance of physicians was mentioned in many other text passages linked to good care, which were unrelated and expressed prior to the closing interview question, so this limitation is possibly not so significant. It may rather highlight the importance of the physician as role how needs to earn interpersonal trust as representative of the system. The final limitation is that the original data collection included interviews with men and women with differing times since diagnosis, which might have influenced patients’ memories about good care. Trust as a ‘leap of faith’, Hillen et al^8^, ^20^, ^30^ following Balkrishnan et al^30^ and Hall et al^8^ have argued, needs to be given before one engages in treatments whereas satisfaction is an evaluation afterwards. While actual activities are prone to severe memory bias, attitudes and emotions remain stable over time. This may be true also for the elements of satisfactory care experiences we have identified in our study.

## CONFLICT OF INTEREST

The authors declare no conflict of interest.

## AUTHOR CONTRIBUTIONS

CH and JMN developed and conducted the DIPEx oncology study, on which the data analysis of this paper is based. CH planned the current analysis study. SB analysed the data and wrote the first manuscript. CH and SB interpreted the study results. CH and GS discussed the results and findings. All authors discussed and edited the final manuscript.

## ETHICAL APPROVAL

DIPEx oncology was approved by the University of Freiburg ethics committee (EA/247/12) and was reported to the Charité – Universitätsmedizin Berlin ethics committee (EA4/053/12).

## INFORMED CONSENT AND PATIENT DETAILS

We confirm that all patient/personal identifiers have been removed or disguised so that the patient(s)/person(s) described are anonymous and cannot be identified through the details of the stories.

## Supporting information

Supplementary Material

## Data Availability

Due to data protection restrictions, additional data are not available.
