# Supplementary material for: Trust, medical expertise and humaneness: A qualitative study on people with cancer’ satisfaction with medical care
Source: Health Expect. 2021 Feb 2;24(2):317–26. doi: 10.1111/hex.13171 (PMC8077133; doi:10.1111/hex.13171)
Supplement: Supplementary file 1 — Supplementary Material [file HEX-24-317-s001.docx]

| **Domain 1**  **Research team and reflexibility** |  |
| --- | --- |
| 1. Interviewer | Page 3 |
| 2. Credentials | Page 3 (Authors listed) |
| 3. Occupation | SB was responsible for the analysis of this study and had no contact with study participants and therefore did not know the interviewees. All involved researchers in the DIPEX project and this project were experienced qualitative researchers working at universities at the time of the DIPEX-project. |
| 4. Gender | Data collection: Interviewer and interviewee had the same gender. Sexual orientation varied with some of the interviewees.  The analysis of this paper was conducted by SB and CH (first and last author listed) |
| 5. Experience and training | The interviewers, (MS and YA) as all other researchers had long-term experience in qualitative research (previous publication Bloedt et al (2018) BMJ open-page 2). |
| **Relationship with participants** |  |
| 6. Relationship established prior the study commencement | Page 2 (previous publication): SB had no contact to the study participants. |
| 7. Participant knowledge of the  interviewer | Name, function, place of employment. No further standardized information was conveyed. However, the interviews were conversational in style. Before and after the interviews conversations often engaged the interviewer and interviewee with each other. |
| 8. Interviewer characteristics | See point 7 and 4 of COREQ. |
| **Domain 2: Study design** |  |
| Theoretical framework |  |
| 9. Methodological orientation & theory | Page 3: We conducted a secondary data analysis using a question-focused approach for all interview material. |
| **Participant selection** |  |
| 10. Sampling | Page 2:  A narrative interview study was conducted with women with a diagnosis of breast cancer and men with a diagnosis of prostate cancer.^18^ The interviews were collected between 2012 and 2013 for the purpose of developing modules for the health information website krankheitserfahrungen.de focusing on people’s experiences of various diseases.  Page 3:  This interview study used maximum variation sampling (with regard to age at interview, age at diagnosis, treatment, course of disease, socio-demographic factors) to include as many different aspects of experiences with breast or prostate cancer as possible.^20,21^  Interview participants were sought from different parts of Germany with the help of the research team, self-help groups, health professionals, primary care clinics and rehabilitation centres. |
| 11. Method of approach | Potential interview partners were approached through contacts of the research team or the organizations mentioned under point 10. |
| 12. Sample Size | Page 4: The sample consisted out of 42 men and 43 women with prostate or breast cancer respectively. |
| 13. Non-participation | Narrative interview data from women with a breast cancer and men with prostate diagnosis were selected based on maximum variation sampling (with regards to age at interview, age at diagnosis, treatment, course of disease, sociodemographic factors) with the aim to include as many different aspects of experience as possible. Since we had a targeted approach it is not possible to say how many potential participants did not participate. |
| 14. Setting of data collection | Page 3/4: Interviewees decided where they wanted to conduct the interview. Interviews were either video- or audio-recorded. |
| 15. Presence of non-participants | No one else was present |
| 16. Description of sample | Page 5: The sample consisted of 43 women with a diagnosis of breast cancer and 42 men with a diagnosis of prostate cancer. The socio-demographics of the interviewees are published elsewhere^11.^. Age and time since diagnosis varied among participants. The age range at diagnosis was 25-71 years in the female sample and 47-74 in the male sample. Time since diagnosis varied from 5 months to 21/15 years for women with a breast cancer diagnosis and men with a prostate cancer diagnosis respectively. |
| **Data collection** |  |
| 17. Interview guide | Page 4: All interviews begun with the same question with the aim to start a narration: “Can you tell me how your life was when you first became aware of the signs/symptoms of cancer and how it went from there. Please take your time and tell me how one thing led to another.” This initial narrative period varied in time depending on the interviewee. Follow-up questions were then asked to elicit all relevant aspects of the potential illness experience. Follow-up questions were based on the initial information provided by the interviewee and included further questions based on a literature review. Themes included were diagnosis process, treatments, information seeking, family and partnership, communication, help and support and living with a cancer diagnosis. The interviews ended with an open question asking what message the interviewee would want to give to fellow patients as well as to physicians. |
| 18. Repeat interviews | No repeat interviews were recorded. |
| 19. Audio/visual recording | Page 3: Interviews were either audio- or video-recorded based on the preference of the interview partners. |
| 20. Field notes | Interviewers wrote a protocol after each interview. |
| 21. Duration | Interview length overall was dependent on interviewee’s which or interest to share their experiences and lasted between half an hour to two hours. One interview took longer. |
| 22. Data saturation | Page 4 states:  This process was complete following the analysis of eight additional interviews. The remaining 25 interviews were read in detail, but this did not further change the core category.  Fourteen more interviews were coded until data saturation was achieved. The remaining 24 interviews added no additional elements or factors. |
| 23. Transcripts returned | The transcripts were given to interviewees to read, correct and agree for their scientific use and presentation on the website. |
| **Domain 3: analysis and findings** |  |
| **Data analysis** |  |
| 24. Number of data coders | Page 4 SB coded the interviews.  Categories and associated text passages relating to satisfaction/dissatisfaction were discussed. The analysis for the two disease entities were discussed in team meeting between the last author (CH) and SB. |
| 25. Description of coding tree | Page 4: Codes related to care were then organised into more conceptual categories according to the attributes displayed relating to satisfaction/dissatisfaction or good/bad care experiences. Categories and associated text passages relating to satisfaction/dissatisfaction were discussed (CH and SB) and grouped into elements and factors of good care. One core category emerged, around which other categories that had been developed from the initial codes could be grouped, thus creating first the factors, than the associated elements associated with care experiences. As analysis pursued factors and elements were refined and further condensed. |
| 26. Derivation of themes | Themes were derived from the data. |
| 27. Software | Based on analysis of the interview data which was entered into MAXQDA for management and data management presented on krankheitserfahrungen.de. |
| 28. Participant checking | Participants did not provide feedback on the findings. |
| **Reporting** |  |
| 29. Quotations presented | page 6-10 |
| 30. Data and findings consistent | Page 5-11. Data and findings are consistent. |
| 31. Clarity of major themes | Major themes are clearly presented on page 5-12. |
| 32. Clarity of minor themes | Minor themes are clearly presented on page 5-12. |
